# The Effects of Low Dose Naltrexone on Opioid Induced Hyperalgesia and Fibromyalgia

**DOI:** 10.3389/fpsyt.2021.593842

**Published:** 2021-02-16

**Authors:** Daniel Jackson, Sunita Singh, Yanli Zhang-James, Stephen Faraone, Brian Johnson

**Affiliations:** ^1^Department of Psychiatry, SUNY Upstate Medical University, Syracuse, NY, United States; ^2^College of Medicine, SUNY Upstate Medical University, Syracuse, NY, United States

**Keywords:** opioid induced hyperalgesia, chronic pain, fibromyalgia, opioid use disorder, low dose naltrexone

## Abstract

**Objectives:** While opioids temporarily alleviate pain, the overshoot of balancing pain drivers may increase pain, leading to opioid induced hyperalgesia (OIH). Our goal was to find out what chronic opioid treatment does to pain tolerance as measured by the cold pressor test (CPT), an objective measure of pain tolerance, and to find an alternative effective treatment for chronic pain and FM.

**Materials and Methods:** The setting was an academic addiction medicine service that has an embedded pain service. Patients had routine clinical care starting with an evaluation that included assessment of medical and psychiatric conditions. Participants were 55 patients with OIH and 21 patients with fibromyalgia; all had at least two CPTs. Treatment included a single dose of buprenorphine for detoxification. In this open-label case series, patients were treated with low dose naltrexone (LDN), a pure opioid receptor antagonist that, we hypothesize, treats OIH and FM by restoring endogenous opioid tone.

**Results:** Comparing initial and last CPT times, those with OIH more than quadrupled their pain tolerance, and those with FM doubled theirs. This improved pain tolerance for OIH and FM was statistically significant (*p* < 0.0001 and *p* = 0.003, respectively) and had a large effect size (*r* = 0.82 and *r* = 0.63, respectively).

**Discussion:** Results suggest that patients on chronic opioid therapy should have pain tolerance measured by CPT with detoxification and LDN provided to correct opioid induced hyperalgesia if found. FM may also be treated with LDN. The main limitation of the findings was lack of a randomized control group treated with placebo.

## Introduction

The opioid epidemic has long entered the public consciousness. Three hundred eighty-three thousand and ninety-one deaths from overdose in the US during 2001 to 2017 has punctuated this awareness (1). Despite a greater appreciation of these ramifications, the prescription of opioids for chronic pain continues. The irony is that opioids worsen pain during the course of long-term use. This phenomenon, opioid induced hyperalgesia (OIH), is the “state of nociceptive sensitization caused by exposure to opioids” (2). OIH's prevalence and optimal management have not been agreed upon (3), and a multitude of compensatory/allostatic changes have been proposed as mechanisms for nociceptive sensitization and mu-opioid receptor desensitization (2, 4–9). OIH leads to a vicious cycle of increasing doses of opioids while increasing pain (6, 10). Such an alteration and dysfunction of the endogenous opioid system is brought about by exogenous opioid use.

Understanding the deleterious effects from chronic exogenous opioid exposure on the endogenous opioid system informs the understanding of fibromyalgia (FM), a syndrome of chronic pain that is diffuse yet accentuated at multiple tender points along with other somatic and cognitive symptoms (11–18). The endogenous opioid system has been hypothesized to play a role in FM, thus joining the numerous and controversial factors considered in FM's pathophysiology (17, 19, 20). Cerebrospinal fluid (CSF) studies of FM patients show elevated kappa-opioid peptide, dynorphin, and met-enkephalin-Arg-Phe, suggesting receptor modulation and desensitization (21, 22). There is significantly decreased mu-opioid binding in the bilateral nucleus accumbens, left amygdala, and right anterior dorsal cingulate on PET scan (23).

We conceptualize FM's alterations in the endogenous opioid system involving an autoimmune process (20). Mu-opioid receptor dysfunction from an autoimmune process may cause increased endogenous opioids produced in an attempt to maintain homeostasis. Ultimately, this mechanism cannot compensate for the diminished binding of mu-opioid receptors, resulting in brain-mediated pain experienced by patients as occurring diffusely over the body.

Given these considerations of the endogenous opioid system in OIH and FM, we present a case series to demonstrate the effect of low dose naltrexone (LDN) on pain tolerance in OIH and FM. Naltrexone's antagonism at mu, kappa, delta, and orphanin FQ/nociceptin opioid receptors and at opioid growth factor receptor (OGFr) induces a variety of cellular responses at different doses (19, 24–38). We submit that the use of low doses, up to 4.5 mg twice a day, of naltrexone restores endogenous opioid tone in OIH and improves it in FM. While the use of opioid antagonists to exert analgesic effects is not a new concept, there is still a dearth of clinical research that investigates such proposed effects in patients. This report of a case series may not elucidate the exact mechanisms underlying the effects of LDN, but we believe that the pilot data is of some interest given the widespread use of opioid medications for chronic pain and the lack of efficacious treatments for FM.

## Methods

### Setting

A pain service is embedded in the Addiction Medicine Service at the State University of New York Upstate Medical University to evaluate pain complaints in patients with comorbid opioid use disorder. Patients are generally poor: 2/3 of our patients have Medicare or Medicaid insurance. Many are chronically ill with multiple medical and psychiatric diagnoses. Prospective patients are not required to have any diagnosis other than chronic pain prior to evaluation on the pain service. Many addicted patients also have chronic pain. Evaluators include medical, physician assistant and psychiatric nurse practitioner students, neurology, internal medicine and psychiatry residents, and pain medicine and addiction psychiatry fellows—along with senior staff physicians and nurse practitioners. Patients are asked to sign an IRB-approved form for their deidentified information to be used in case series reports. Treatment progress is monitored by joining subjective reports of pain with the cold pressor test (CPT), a validated, objective measure of chronic and experimental pain (39), with good test-retest reliability (40). In addition to transference-focused psychotherapy (41, 42) and holistic medical treatment, patients are treated with LDN.

### Participants

We reviewed all patients who presented for an initial intake between January 2017 and July 2019. There were 786 initial evaluations. Three hundred seventy six had an initial CPT. Seventy six were treated with LDN and had follow-up CPTs. Roughly half of all patients who present to the Addiction Medicine Service are evaluated at the embedded pain service. Of these patients, a smaller proportion have fibromyalgia rather than opioid use disorder, as reflected in the larger number of OIH patients in the study sample. Of the 76 patients treated with LDN and had follow-up CPTs, 55 were diagnosed with OIH, and 21 were diagnosed with FM.

Because the evaluations are complex, and cognitive impairment is common, we require that every new patient bring a sober support person. The support person is present for the evaluation and discussion of diagnoses, proposed treatments, and whether to engage in treatment on the service. Support persons from a prior case series had average CPT of 113 seconds (43). In that study, we chose the support persons as our control group because they were close in nature to our patient population by virtue of having been asked by our patients to participate. Before testing the support persons, we asked if they had recent exposure to nicotine, opioids or cannabis. Only support persons without these potentially pain tolerance-altering exposures were used.

### Evaluation

The services have a holistic nature. We start with the chief complaint and history of present illness, then the psychiatric, medical, family and social histories. A comprehensive substance use history is taken on alcohol, cannabis, nicotine, cocaine, amphetamine, benzodiazepine and opioid use. A Hamilton Rating Scale for Depression, a Modified Mini-Mental State Examination and a FACES Pain Scale (FPS) are recorded.

We use screens for common comorbid disorders. The Adult ADHD Self Report Scale for attention deficit hyperactivity disorder (ADHD) is given, followed by a DSM5 interview if ADHD is suspected. The Structured Clinical Interview for DSM5 (SCID2) checklist is used to screen for borderline personality disorder. While ADHD, borderline personality and depressive disorders are unusual in pain patients, they are present in about half of our opioid use disorder patients (44). A physical examination is part of every evaluation. If chronic pain is present, the examination focuses on the peripheral pain driver. If FM is suspected, the 18 potential tender points are palpated, and the number of tender points is reported.

### Morphine Years

The dose, frequency, and duration of opioid exposure, obtained in the substance history, are rendered as morphine years (MY). A “morphine year” had been described in a previous publication as daily use for a year at 60 morphine milligram equivalents (MME). We had found a positive correlation between MY and the prevalence of depression, ADHD, and borderline personality. MY were higher in younger patients because of the use of illicit opioids, as doses sold by street dealers are about 100 times greater than prescribers. We had found a negative correlation between CPT and MY, with more cumulative opioid use leading to lower CPT, suggesting that opioid exposure causes a steady decrease in pain tolerance (43).

### CPT and OIH

Exposure to opioids almost always caused short CPT. CPT was repeated on follow-up appointments to gauge changes in pain sensitivity. The test was stopped at 180 s for patients that had a high pain tolerance. Changes in pain sensitivity were used to reassess LDN treatment. Patients also reported their pain on initial evaluation via the FACES Pain Scale. On follow-up visits, patients reported if their pain was better, worse, or no change. Patients were diagnosed with OIH if they were experiencing significant pain while on opioids and had a CPT less than two-thirds of the healthy controls' average of 113 s (43).

### Opioid Detoxification

The patient arrives in early withdrawal with symptoms such as gut cramps, anxiety, and increased pain. 8 mg sublingual buprenorphine tablets are taken in front of staff until withdrawal symptoms remit; usually 24 or 32 mg are sufficient. The duration of the “detox” is about 15 min. Patients were sent home with clonidine, hyoscyamine, trazodone, olanzapine, and gabapentin for attenuated withdrawal symptoms. These symptoms and their treatments were explained on a written handout topped by the senior author's cell phone number. Directions were given to “call day or night if you need help” (there are few calls). In addition to pharmacotherapy, patients received psychotherapy daily for the first week and then twice a week until discharge. After buprenorphine administration, LDN was started at 0.1 mg twice a day and titrated with the following schedule:

- 0.2 mg twice a day on day two
- 0.3 mg twice a day on day three
- 0.4 mg twice a day on day four
- 0.5 mg twice a day on day five
- 1.0 mg twice a day for days five and six
- 2.0 mg twice a day for days 7 and 8
- 4.5 twice a day thereafter

Given the difficulty with finding pharmacies that will accommodate this varied dosing, we dissolve a 50 mg pill of naltrexone in 50 ml of water, and we show patients how to use an insulin syringe to draw up 0.1 mg increments. This titration occurred more slowly if there was a return of opioid withdrawal symptoms with increased dosing, understood as if the receptor system was slow to regenerate and therefore not tolerating the increasing doses of LDN. FM patients not currently on opioids started LDN as soon as their treatment plan was agreed upon.

### Statistics

Stata 16 was utilized. The Wilcoxon signed-rank test was used to determine the statistical significance of the change in CPT. Ordinal least squares regression was used to determine if the relationship of change in CPT varied significantly with age, sex, days between first and last measurement, FPS, and MY. Non-parametric statistics were used because the data did not meet the normality assumption.

## Results

Of the 363 patients who had an initial CPT, 76 returned for follow-up and continued treatment with LDN. This reflects the nature of our service; some patients will not return for treatment because:

- They believe that they should be treated with opioids despite short CPTs that are diagnostic of OIH
- Some expect pills to fix their pain exclusively and will not engage in active treatments that include the examination of a lack of self-care during an extended evaluation that requires appearing for further visits
- Some seek a source of opioids and drop out when it becomes apparent that opioids are not part of the treatment

The remaining 20% of patients were a highly motivated group, culled from an intake system that requires active engagement. The results from these 55 OIH and 21 FM patients are summarized in Table 1. The patients varied on the number of follow-up CPTs (ranging from 2 to 4) and the interval between their follow-up CPTs. Therefore, the change in their pain tolerance was tabulated from the difference in the last CPT from their initial CPT. The time between their initial and final CPT was also recorded.

**Table 1:** Averages in demographics, change in CPT, and FACES Pain Scale for OIH and FM patients, with 95% confidence interval in brackets.

	**OIH (*N* = 55)**	**FM (*N* = 21)**
Age (years)	53.8 [49.66, 57.94]	43.48 [37.1, 49.85]
Sex	50.9% Female [n/a]	90.5% Female [n/a]
Change (seconds) from 1st and last CPT	83.07 [64.61, 101.5]	16.05 [3.415, 28.68]
Days b/w 1st and last CPT	89.78 [63.3, 116.3]	48.14 [36.45, 59.83]
FACES Pain Scale on initial evaluation	5.425 [4.642, 6.207]	6.786 [6.101, 7.471]

**Figure 1:**
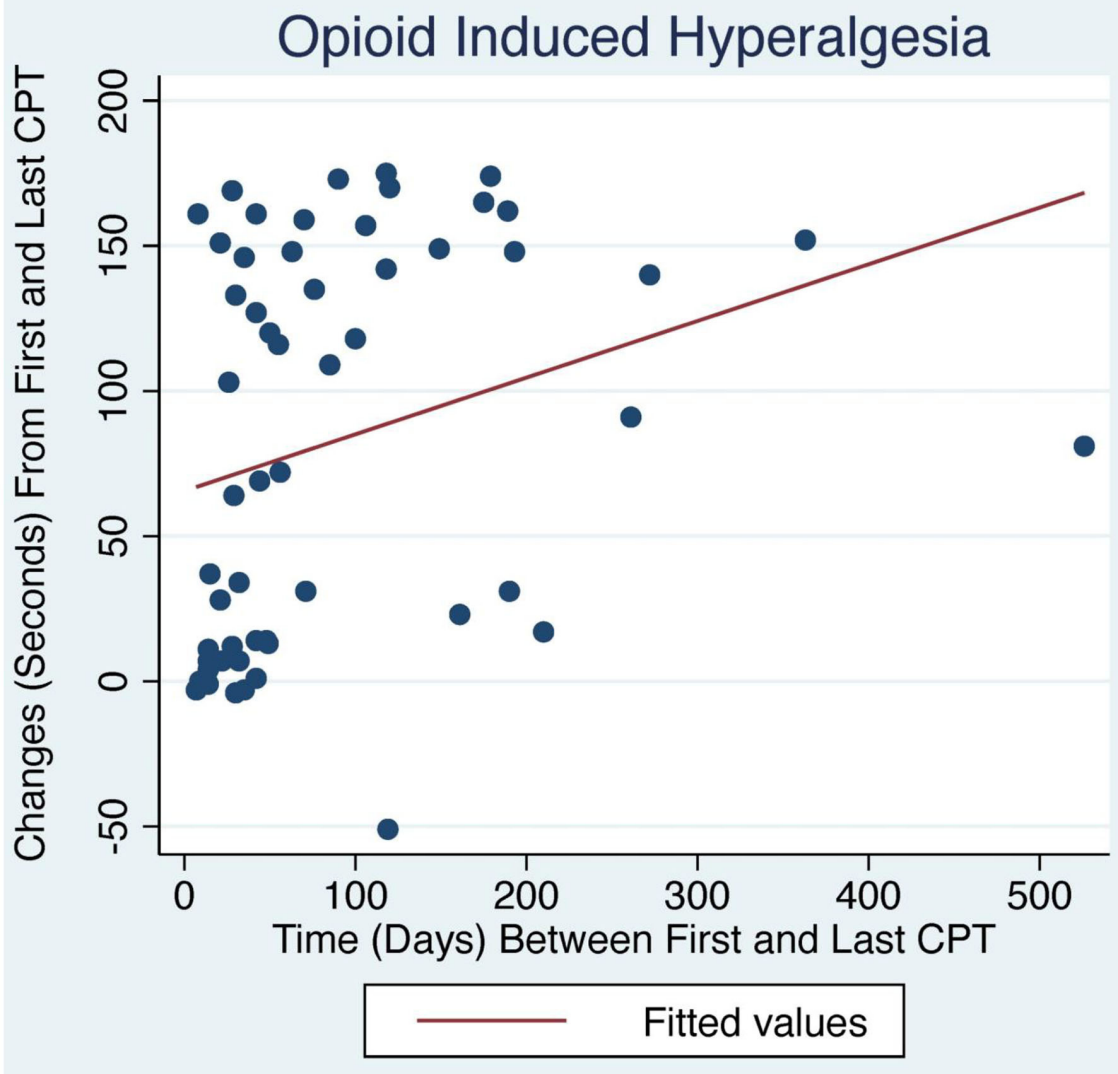
Change in CPT over time with LDN treatment. OIH patients treated with LDN showed a positive relationship between change in CPT and number of days between first and last CPT measurement (*p* < 0.04).

**Figure 2:**
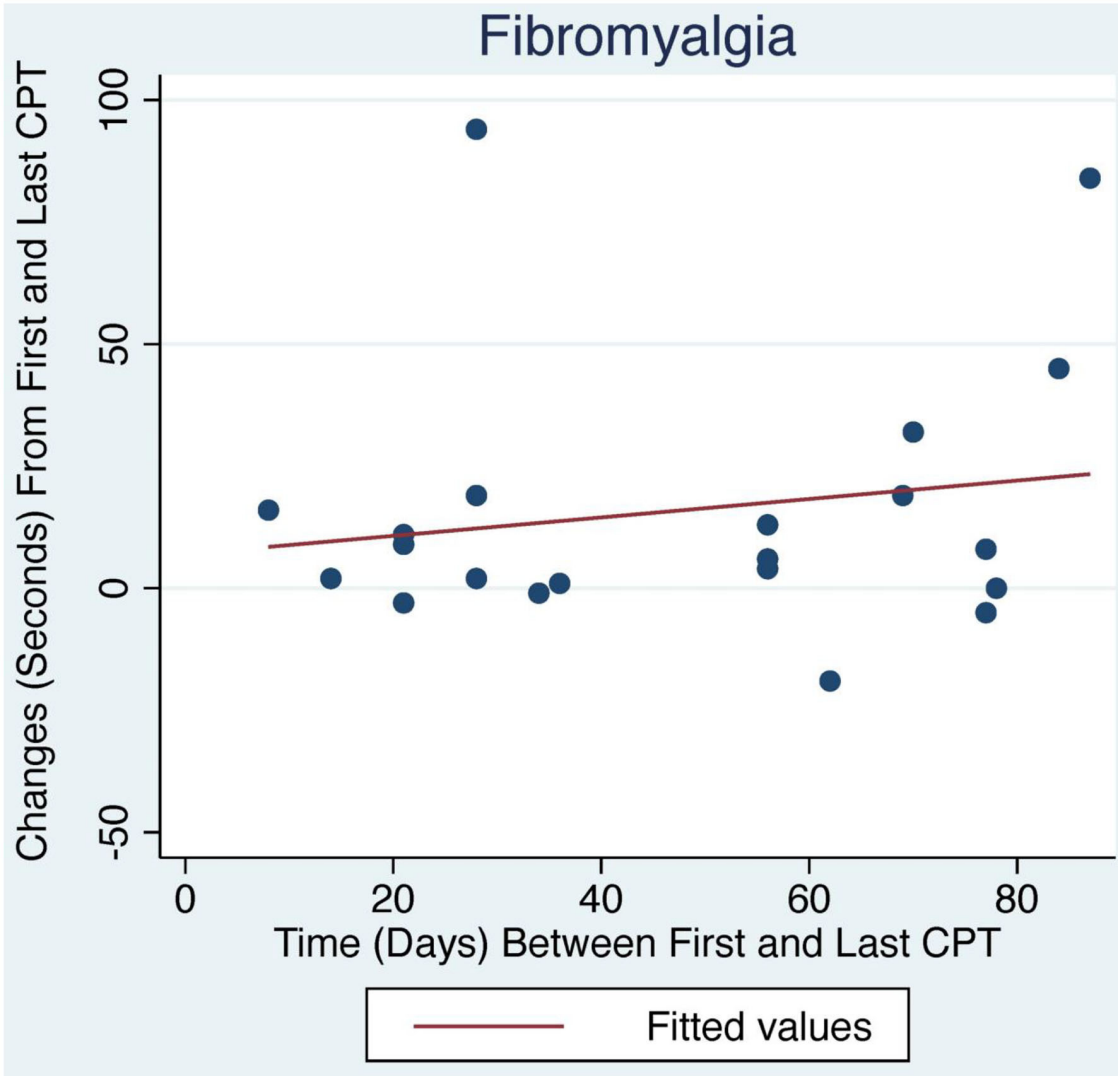
Change in CPT over time with LDN treatment for FM patients. The relationship between change in CPT and number of days between first and last CPT measurement did not reach statistical significance.

The average initial CPT was low for both OIH (24 s) and FM (14 s) when considering the control group from a prior case series had an initial pain tolerance of 113 s (43). The patients' low CPTs were mirrored by their high subjective pain ratings, as the FACES Pain Scale at the initial evaluation averaged 5.4/10 for OIH and 6.8/10 for FM. OIH patients demonstrated a more robust change in their CPT over time as well as having more days between their initial and final CPT compared to FM patients. OIH patients averaged an improvement of 83 s in their pain tolerance (*p* < 0.0001). FM patients exhibited an increase in their pain tolerance of 16 s (*p* < 0.003). The effect sizes were substantial (*r* = 0.82 for OIH and *r* = 0.63 for FM). After their initial CPT, OIH patients averaged 3 months before they completed their last repeat CPT. FM patients averaged 7 weeks between their initial and final CPT. Only in OIH was there a statistically significant relationship between the change in CPT and the number of days between the first and last CPT measurement (*p* < 0.04, see Figures 1, 2). The change in CPT was not significantly correlated, in either diagnosis, with age, sex, FPS, or MY.

## Discussion

Patients maintained on opioids for chronic pain presented with an average initial CPT of 24 s and FPS of 5.4/10, underscoring their diminished pain tolerance when compared to a prior control group's 113 s average (43). However, 3 months of treatment with LDN more than quadrupled OIH patients' pain tolerance; their average of 107 s at their last CPT suggests a restoration of their endogenous opioid tone. The small though significant correlation of the improvement in pain tolerance with number of days between the first and last CPT may indicate that the endogenous opioid system needs time to normalize, perhaps 3 months on average. FM patients started with more pain compared to OIH patients with FPS of 6.8 and initial CPT of 14 s. They were comprised of 90% women compared to the equal sex distribution in OIH. Their CPT responded more sluggishly over seven weeks to 30 s, coinciding with FM patients typically reporting an improvement in pain but not complete resolution.

What accounts for the greater magnitude of response to LDN found in OIH compared to FM? While we propose that the endogenous opioid system is integral to the pathophysiology of both conditions, the mechanisms by which LDN may restore endogenous opioid tone remains largely a mystery, as varying doses and binding durations may produce different effects at each of the opioid receptor types (28, 34, 36). The restoration of endogenous opioid tone may have primacy in correcting OIH (19, 20), but FM may have a neuroimmunological component distinct from OIH. LDN may influence neuroimmunomodulation by intermittent blockade of opioid growth factor receptor (OGFr). The transient blockade of OGFr by naltrexone increases levels of OGF (37). In animal studies, the increase of OGF is associated with the decrease of neuronal damage, inflammation, and proliferation of T and B cells (29, 45–47); however, LDN may correct a disruption to the OGF-OGFr axis rather than simply increasing enkephalin levels alone. Prior findings of a possible increase in endogenous opioid levels in FM (21, 22) could be a compensatory response to receptor degradation (20), but LDN may ameliorate such receptor degradation through the upregulation of OGFr. This may be especially true for those in which signs of an active inflammatory process are present, such as increased glial activation along with increased cytokines in the CSF (and in some cases, plasma) in FM patients (48–50). Since erythrocyte sedimentation rate levels can predict the therapeutic response to LDN (51), the variation of an inflammatory component, with its modulation via the OGF-OGFr axis, may indicate why FM patients have a significant but smaller response to LDN compared to OIH patients. Until markers for FM can be identified and targeted for treatment, LDN can alleviate most patients' pain, cognitive, affective, and systemic symptoms, all products of low opioid tone likely caused by opioid receptor degradation (20).

Alterations in the affective processing of pain in FM and OIH (52, 53) may also account for the varying baseline pain tolerance and response to LDN. Patients with FM have increased sympathetic nervous system activity compared to healthy controls (48), and decreased mu-opioid binding potential occurs in areas associated with the affective processing of pain (23). Functional imaging and gray-matter volume studies of FM patients show differences in areas implicated in the emotional modulation of pain, but results vary when controlling for comorbid mood disorders (53–55). However, similar findings in functional connectivity alterations have been found in those with long-term opioid use (56), and the emerging research on opioid receptors and mood may reveal a means for which LDN treats the opponent process' effects on mood that increase relapse and worsen pain (56–59). Therefore, the neuroimmunological component may be the best explanation at this time to account for the differences in improvement between OIH and FM.

Given these encouraging results, what are the implications for future research as well as the current status of the use of opioids in treating chronic pain? The brain rebels against chronic opioid treatment by the opponent process (10, 60, 61), increasing pain drivers such as glutamate, dynorphin, corticotrophin releasing factor, and substance P. Clinicians and patients escalate the doses of opioids to temporarily meet the body's homeostatic response. This seems to continue to diminish the brain's endogenous opioid system, amplifying the central response to peripheral pain drivers. In addition to this worsening of pain, chronic use of opioids poses risks such as falls, hypogonadism, and constipation. The chronic use of opioids may also instigate opioid use disorder, as three-quarters of heroin users begin with prescription opioids (62, 63). Finally, a seldomly-noted side-effect of chronic opioid treatment is flattened relatedness to others. Our understanding of this phenomenon has to do with the endogenous opioid system's regulation of closeness with others (20). However, should opioids be discontinued in a patient with chronic pain via a slow taper, the opponent process is left unchecked, instigating prolonged withdrawal. This is because the brain has made allostatic change to accommodate chronic opioid treatment, increasing pain drivers. The detoxification process described above provides an alternative to tapering, and the restoration of endogenous opioid tone by LDN leads to restoration of relatedness. Support persons make comments such as, “I have the woman/man I married back!” Such encouraging results from this pilot data are of interest given the continued use of opioids for chronic pain and the lack of efficacious treatments for FM, and they indicate the need for double blind, randomized-controlled trials.

## Limitations

This is a chart review study of the cold pressor test in a variety of patients presenting for addiction and/or pain treatment. The topic and the concept of the study are certainly of interest, but the methodology is challenging, as the CPT data were derived from charts and was not delivered in a standardized fashion (in terms of timing, etc). Ideally, one would need demographically matched groups of controls, patients with chronic pain not on opioids, patients with chronic pain on opioids without addiction, and patients with chronic pain who are on opioids who are addicted. Patients who had no history of opioid exposure were so unusual that we were not able to find enough subjects to construct such a control group.

Since subjects were not randomized to experimental and control groups, the potential of a placebo effect could not be evaluated. However, it may be that placebo responses do not significantly affect cold pressor pain. Placebo effects are shown to be greater in clinical pain (such as low-back pain) rather than experimental pain (CPT) (64). Since our results were drawn from multiple CPTs, this may raise the possibility of a conditioned placebo response as well, yet it has been theorized that conditioned placebo responses may not be found for several reasons (65). Factors including type of pain, expectation of relief from naltrexone, expectation of pain from CPT, and the sex of patients and of the evaluators administering the test create a multitude of variables for potential placebo and nocebo responses. The evaluation of all of these factors are beyond the scope of this paper as different pathways without a unifying model are likely responsible for placebo effects when they are present (66). Moreover, if dopaminergic pathways are responsible for the placebo response (67–69), then the usurping of these pathways by addiction may alter the placebo response itself.

It is possible that patients who were experiencing positive results from the treatment remained in treatment long enough to have a second CPT, while non-responders dropped out, skewing the reported results toward responders. Our titration schedule takes into account the sensitivity of the already diminished endogenous opioid system of our patients. Recent findings suggest a dose-response relationship to LDN among FM patients (70); it is possible that our titration schedule mitigates adverse effects but can lead to dropout if an effective dose is not reached quickly enough for certain patients. Finally, given that those with opioid addiction and FM have alterations in the affective processing of pain (23, 54, 55, 58, 61, 71, 72), multiple treatment modalities that may alter affective processing could confound the effects of LDN. Psychotherapy and medications, in addition to LDN, may have a “synergistic effect on recovery of endogenous opioid tone” (73). Medications used to alleviate withdrawal symptoms may affect pain. Controlling for these variables in an RCT is warranted.

## Conclusion

By constantly using patient feedback, we have been able to discover how to detoxify patients from opioids as an easy outpatient procedure, assess pain tolerance with the cold pressor test, and ameliorate opioid induced hyperalgesia with low dose naltrexone. These are all innovative procedures. The weakness of our report is the lack of randomized control groups.

Routine use of CPT is helpful in diagnosing OIH. It helps patients see that using opioids for chronic pain treatment increases pain. Opioids have a high prevalence of risks, including death from accidental overdose, iatrogenic addiction, unrelatedness, falls, and constipation.

FM mimics OIH; its symptoms are congruent possibly because it is an autoimmune disease that also reduces CNS pain-damping opioid tone (20). Detoxification, attention to underlying emotional issues, and LDN can make a substantial difference for patients as shown in our case series report. Further investigation via double blind, randomized-controlled trials of LDN is indicated.

## Data Availability Statement

The raw data supporting the conclusions of this article will be made available by the authors, without undue reservation.

## Author Contributions

DJ wrote the first draft. All other authors contributed intellectually to the manuscript.

## Conflict of Interest

The authors declare that the research was conducted in the absence of any commercial or financial relationships that could be construed as a potential conflict of interest.
